# Solid-State Fermented Pineapple Peel: A Novel Food Ingredient with Antioxidant and Anti-Inflammatory Properties

**DOI:** 10.3390/foods12224162

**Published:** 2023-11-17

**Authors:** Erika Ortega-Hernández, Lucio Martinez-Alvarado, Beatriz A. Acosta-Estrada, Marilena Antunes-Ricardo

**Affiliations:** 1 Tecnologico de Monterrey, Escuela de Ingeniería y Ciencias, Centro de Biotecnología-FEMSA, Ave. Eugenio Garza Sada 2501 Sur, Monterrey CP 64849, Mexico; 2 Tecnologico de Monterrey, Institute for Obesity Research, Ave. Eugenio Garza Sada 2501 Sur, Monterrey CP 64849, Mexico

**Keywords:** solid-state fermentation, pineapple peel, phenolic compounds, *L. plantarum*, *L. rhamnosus*, *A. oryzae*, antioxidant capacity, anti-inflammatory activity

## Abstract

It has been reported that pineapple (*Ananas comosus*) contains healthy nutrients and phytochemicals associated with antioxidant and anti-inflammatory capacities. However, a substantial amount of pineapple residue is produced due to a lack of valorization applications at the industrial scale, resulting in the loss of valuable nutrients. Solid-state fermentation (SSF) is proposed as an innovative strategy to enhance the release of bound phenolics from pineapple residues. In this work, the effects of SSF of pineapple peels with *Lactobacillus plantarum*, *Lactobacillus rhamnosus,* and *Aspergillus oryzae* on the release of phenolic compounds and their antioxidant and anti-inflammatory activities were evaluated, respectively. Pineapple peel extracts after SSF showed an increase in the release of phenolic compounds (248.11% with *L. plantarum*, 182% with *A. oryzae*, and 180.10% with *L. rhamnosus*), which led to an increase in the cellular antioxidant (81.94% with *L. rhamnosus*) and anti-inflammatory potential (nitric oxide inhibition of 62% with *L. rhamnosus*) compared to non-fermented extracts. Therefore, SSF of pineapple peels with *L. plantarum*, *L. rhamnosus,* and *A. oryzae* thrives as a new approach for the production of secondary metabolites with remarkable biological benefits, which can be the precursors for novel biofortified and nutraceutical-enriched foods that meet the needs of the most demanding and health-conscious consumers.

## 1. Introduction

Waste disposal is a significant issue for many agro-industries since it is highly susceptible to microbial decomposition and causes substantial environmental problems. According to the Food and Agriculture Organization of the United Nations (FAO), just around 14% of the food produced was lost from the post-harvest stage in the world [1]. Using agro-industrial waste by conversion into value-added products may be an excellent solution to environmental pollution [2]. 

Pineapple (*Ananas comosus*), the only edible member of the family Bromeliaceae, is widely cultivated in several tropical countries, including the Philippines, Thailand, Indonesia, Malaysia, Kenya, India, China, and South America [3]. Pineapple is usually consumed as fresh pulp or processed into different products, including jams, purees, or canned juices [4]. However, about 80% of the total fruit weight in the form of the crown, outer peel, and core is discarded, causing a waste disposal problem of about 22.5 million tons of pineapple annually [5,6]. These residues are rich in cellulose, hemicellulose, and phenolic compounds, which have been recognized as antioxidants and for preventing chronic inflammation, cardiovascular disease (CVD), cancer, and diabetes [7].

In addition, pineapple residues are a source of polyphenols with strong antioxidant activities. Most phenolic compounds occur primarily in conjugated form, bound to the matrix, making it difficult to extract or liberate them [7]. Since the resonance stabilization of free radicals depends on the presence of free hydroxyl groups on the phenolic rings, these conjugations diminish their capacity to function as effective antioxidants [8]. 

The pretreatment of agro-industrial residues with solid-state fermentation (SSF) system technology could improve the recovery of phenolic compounds through the hydrolysis of these conjugates with microorganism-produced degrading enzymes [9]. SSF consists of using moist substrates for a microbial culture in the near absence of available water [10]. For example, *Lactobacillus plantarum* and *Lactobacillus rhamnosus* are microorganisms that have been used in many SSF studies because of their ability to synthesize hydrolytic enzymes [11,12,13,14].

*Aspergillus oryzae* is an important food-grade filamentous fungus that has been used in fermentation technologies for the preparation of traditional Asian fermented foods, such as sake, miso, and shoyu [15]. In SSF processes, high amounts of β-glucosidase can be produced, which plays a crucial role in the hydrolysis of phenolic glycosides [16].

Since studies on using pineapple waste as a substrate in SSF systems for producing added-value compounds are limited, further research is necessary. 

Therefore, this study aims to evaluate the feasibility of pineapple residue as a substrate for producing phenolic compounds with antioxidant and anti-inflammatory activities by *L. plantarum*, *L. rhamnosus*, and *A. oryzae* via SSF.

## 2. Materials and Methods

### 2.1. Chemicals

Methanol (HPLC grade), water (HPLC grade), and sodium chloride (NaCl) were obtained from CTR, S.A. de C.V. (Monterrey, NL, Mexico). GIBCO (Carlsbad, CA, USA) supplied Dulbecco Modified Eagle Medium (DMEM), Penicillin-Streptomycin antibiotic (Pen-Strep), fetal bovine serum, and phosphate-buffered saline (PBS, pH 7.4). Griess Reagent System and CellTiter 96 AQueous One Solution Cell Proliferation Assay kits were purchased from Promega (G2930, Madison, WI, USA). IL-2, IL-6, IL-1β, and TNF-α MILLIPLEX MAP Mouse Cytokine/Chemokine panel Human/Mouse kit was obtained from Millipore (Billerica, MA, USA). Total COX-2 DuoSet IC ELISA and Mouse IL-10 ELISA were purchased from R&D Systems (Minneapolis, MN, USA). The other chemicals were obtained from Sigma-Aldrich Co. (St. Louis, MO, USA).

### 2.2. Plant Material

Pineapple peels of the pineapple (*Ananas comosus*) Golden MD2 variety obtained from the local market (Monterrey, México) during June 2021 were washed, freeze-dried (LABCONCO, Kansas City, MO, USA), ground into a powder, sifted, and stored at −80 °C for further analysis. 

### 2.3. Pineapple Peel Bromatological Analysis and Chemical Composition

Pineapple peels were analyzed using the Mexican codex to determine their total reducing sugars (NOM-086-SSA1-1994), calories (NOM-086-SSA1-1994), available carbohydrates (NOM-086-SSA1-1994), ashes (NMX-F-607-NORMEX-2013), ether extract (NOM-086-SSA1-1994), dietary fiber (NOM-086-SSA1-1994), moisture (NOM-086-SSA1-1994), and protein content (NMX-F-608-NORMEX-2011). 

### 2.4. Preparation of Bacterial Inoculums

Probiotic strains *Lactobacillus plantarum* 299v (DSMZ 9843) and *Lactobacillus rhamnosus* GG (ATCC 7469) were obtained from the German Collection of Microorganisms and Cell Cultures (DSMZ, Braunschweig, Germany) and the American Type Culture Collection (ATCC, Manassas, VA, USA), respectively, and were activated following the method reported by Yan et al. [17]. Bacterial strains were cultured in MRS (Man, Rogosa, and Sharpe) broth at 37 °C for 24 h. Bacterial strains were subcultured two more times, repeating the last step. Then, strains were centrifuged (4000× *g*, 5 min, 4 °C), washed using a sterile 0.85% *w*/*v* NaCl solution, and adjusted to a final concentration of 7 log colony-forming unit CFU/mL. Cell density was measured by optical density (OD) using a spectrophotometer (Genesys 10S, Thermo Scientific, Waltham, MA, USA) at 600 nm.

### 2.5. Preparation of Fungal Inoculum

*A. oryzae* spores (ATCC 22788) obtained from the American Type Culture Collection (ATCC, Manassas, VA, USA) were inoculated following the method reported by Villasante et al. [18]. Spores were cultured in potato dextrose agar and incubated at 30 °C for 5 days. Spores were collected using 10 mL of 0.1% *v*/*v* Tween 80 solution with distilled water and counted using a hemocytometer (Bright-Line, Hausser Scientific, Horshara, PA, USA) in a microscope (Olympus CK2, Marshall Scientific, Hampton, NH, USA). Spores were diluted to reach a concentration of 5 log spores/mL. 

### 2.6. Solid-State Fermentation with L. plantarum and L. rhamnosus

For the solid-state fermentation of pineapple peels using lactic acid bacteria, 5 mL of *L. plantarum* and *L. rhamnosus* 0.85% sodium chloride (NaCl) solution was inoculated into 5 g of pineapple peels previously sterilized with ultraviolet light for 30 min to ensure the absence of other bacteria and fungi. Water activity (Aw) of *L. plantarum* and *L. rhamnosus* was determined using an AquaLab Dew Point Water Activity Meter 4TE (Decagon Devices, Pullman, WA, USA) at 24 °C. Moisture content during fermentation was 88%. 

The fermentation process was performed in triplicate for 5 days at 37 °C in an incubator (Shel Lab 1535, Sheldon Manufacturing Inc., Cornelius, OR, USA). The initial pH of fermented pineapple was adjusted to 4. Samples were collected at 24 h intervals and stored at −80 °C for analysis of phenolic compounds and bioactivity. 

### 2.7. Solid-State Fermentation with A. oryzae

For the solid-state fermentation of pineapple peels using *A. oryzae*, 5 mL of 0.1% Tween 20 solution was inoculated into 5 g of freeze-dried pineapple peels previously sterilized with ultraviolet light for 30 min [18]. Water activity (Aw) of *A. oryzae* was determined using an AquaLab Dew Point Water Activity Meter 4TE (Decagon Devices, Pullman, WA, USA) at 24 °C. Moisture content during fermentation was 88%. 

The fermentation process was performed in triplicate for 5 days at 30 °C in an incubator (Shel Lab 1535, Sheldon Manufacturing Inc., Cornelius, OR, USA). The initial pH of fermented pineapple was adjusted to 4. Samples were collected at 24 h intervals and stored at −80 °C for analysis of phenolic compounds and bioactivity.

### 2.8. Phytochemical Analysis

#### 2.8.1. Phenolic Compounds Extraction

After the solid-state fermentation of pineapple peels, phenolic compound extraction was performed using a slightly modified protocol from Acosta-Estrada et al. [19]. Briefly, a methanol (80%) solution was added to previously freeze-dried solid-state fermentation samples in a proportion of 1:20 (*w*/*v*). Then, samples were vortexed for 1 min and stirred at 250 rpm for 10 min at 25 °C using an orbital shaker (Incubator 3500I, VWR International, Radnor, PA, USA). Samples were centrifuged (3000× *g*, 10 min, 4 °C), and the supernatant was recovered, freeze-dried (LABCONCO, Kansas City, MO, USA), and stored at −80 °C for further analysis.

#### 2.8.2. HPLC-DAD Analysis of Phenolic Compounds

Fermented pineapple peel extracts (FPPE) were solubilized in 80% methanol and analyzed through high-performance liquid chromatography with diode-array detection (HPLC-DAD) according to the method reported by Steingass et al. [20]. Analyses of phenolic compounds were performed using a high-performance liquid chromatography system coupled with a diode-array detector (HPLC-DAD) (1260 Infinity, Agilent Technologies, Santa Clara, CA, USA). The column used to separate phenolic compounds was a Luna C18(2) Phenomenex™ (250 × 4.6 mm, 5 μm particle size). Water (A) and methanol containing 1% (*v*/*v*) formic acid (B) were used as the mobile phase. The following gradients were used: 5 to 40% B (35 min), 40 to 70% B (15 min), 70 to 100% B (2 min), and isocratic hold at 100% B (3 min). The column was flushed back to 5% B (2 min) and held isocratically for 8 min. The total run time was 65 min at a flow rate of 0.8 mL/min and an oven temperature of 30 °C. The injection volume was 10 μL. The detection wavelengths were set to 280, 320, and 360 nm. Chromatographic data were processed with OpenLAB CDS ChemStation software version 1.8 (Agilent Technologies, Santa Clara, CA, USA).

Mass spectra of phenolic compounds in FPPE were identified according to the method reported by Steingass et al. [20], following the abovementioned method. It was performed using liquid chromatography time-of-flight mass spectrometry (LC/MS-TOF) coupled with electrospray ionization (ESI) (Agilent 1100 system, Agilent Technologies) using the same conditions for the HPLC-DAD analysis. Mass spectra were scanned in a range of *m*/*z* 100–1500 through positive electrospray ionization [ESI (+)]. Nitrogen served as dry gas at a flow rate of 13 L/min and nebulizing gas at a pressure of 45 psi. The gas temperature was set to 350 °C, and the capillary potential was 4000 V. The fragmentation amplitude was set to 120 V. Mass Hunter Software version A.02.00 2005 (Agilent Technologies, Santa Clara, CA, USA). Analyst QS 1.1 Software (Applied Biosystems, Waltham, MA, USA) was used for the identification of compounds present in FPPE. 

The identification of phenolic compounds was based on the retention time, DAD spectra, and their mass-to-charge (*m*/*z*) ratio. Quantification of phenolic compounds was performed using gallic acid, *p*-coumaric acid, ferulic acid, and quercetin as standards. Results were expressed as µg equivalents of each phenolic compound per g of FPPE in dry weight (DW).

### 2.9. 2,2-Diphenyl-1-Picrylhydrazyl (DPPH) Scavenging Activity Assay

DPPH was carried out using the method reported by Ortega-Hernández et al. [21]. Briefly, an aliquot of FPPE (100 µL) at different concentrations (1000 µg/mL, 500 µg/mL, and 250 µg/mL) was added to 100 µL of methanol DPPH (0.1 mM) solution. The solution was incubated for 30 min at 37 °C in dark conditions. After incubation, absorbance was measured at 517 nm. The scavenging activity percentage (%) was calculated using Equation (1):

(1) $$DPPH\;Scavenging\;\left(\%\right)=\frac{A\;blank-A\;sample}{A\;blank}\times 100$$

### 2.10. Lipid Peroxidation Inhibition

The inhibitory effect of FPPE on lipid peroxidation was evaluated in a phospholipid system using the method proposed by Guo et al. [22]. First, lecithin solution (0.25% *w*/*v*, 500 µL) was mixed with 200 µL of FPPE at different concentrations (1000 µg/mL, 500 µg/mL, and 250 µg/mL) and 300 µL of water. Then, 50 µL FeSO_4_ (70 mM) was added, and the reaction solution was incubated at 37 °C for 30 min, followed by 500 µL trichloroacetic acid (TCA) (10% *w*/*v*) and thiobarbituric acid (TBA) (1% *w*/*v*). The mixture was vortexed and incubated at 100 °C for 1 h in an oven, and then centrifuged (10,000× *g*, 5 min, 4 °C) to collect the supernatant. The absorbance of the supernatant was measured at 532 nm. 

Lipid peroxidation inhibition was calculated using Equation (2):

(2) $$Inhibtion\;\left(\%\right)=\frac{A\;blank-A\;sample}{A\;blank}\times 100$$

### 2.11. Biological Activity In Vitro of Fermented Pineapple Peel Extracts (FPPE)

The effect of FPPE on cellular antioxidant activity (CAA), nitric oxide (NOx), and cytokines (IL-1β, IL-2, IL-6, IL-10, TNF-α, and COX-2) production was evaluated.

#### 2.11.1. Cell Culture 

Human colon cells (Caco2) and murine macrophage cells (Raw 264.7) were obtained from the American Tissue Culture Collection (ATCC; Manassas, VA, USA) and cultured in Dulbecco’s Modified Eagle Medium (DMEM-F12) supplemented with 5% fetal bovine serum (FBS), incubated at 37 °C in 5% CO_2_. 

#### 2.11.2. Cellular Antioxidant Capacity Assay

The cellular antioxidant capacity assay was carried out according to the methodology of Ortega-Hernández et al. [23]. Briefly, Caco2 cells were cultured in 96-well plates (5 × 10^4^ cells/well) and allowed to adhere for 24 h. Then, cells were washed with PBS solution (pH 7.4) and treated with 100 μL of FPPE (25 µg/mL) containing DCFH-DA (60 μM). After incubation at 37 °C for 20 min, the treatment solutions were removed, and the cells were washed twice with a PBS solution. Finally, 100 μL of 500 μM AAPH solution was added to each well, except for the blank and negative control wells. Fluorescence emitted at 538 nm with excitation at 485 nm was measured with a microplate reader (Synergy HT, Bio-Tek, Winooski, VM, USA) every 2 min for 90 min at 37 °C. 

The CAA values were calculated using Equation (3):

(3) $$CAA\;Unit=1-(\frac{\int SA}{\int CA})$$

where *∫ SA* is the integrated area under the sample fluorescence versus time curve and *∫ CA* is the integrated area of the control curve.

#### 2.11.3. Cellular Anti-Inflammatory Potential Assay

The anti-inflammatory potential of the extracts was performed using the method proposed by Ortega-Hernández et al. [23]. Raw 264.7 cells were cultured in 96-well plates (5 × 10^4^ cells/well) and allowed to adhere for 24 h. Then, cells were treated with 50 μL of FPPE (25 µg/mL) and incubated for 4 h. Following, half of the wells were stimulated with lipopolysaccharide (LPS) at 1 μg/mL while the other half was used as the control for each sample. After 24 h of incubation, the nitrite concentration in the medium (100 μL) was measured at 550 nm (Synergy HT, Bio-Tek, Winooski, VM, USA). The nitric oxide (NO) production was measured using a nitrite standard curve (1.5–50 µM). 

#### 2.11.4. Measurement of Raw 264.7 Cell Viability

Cell viability was tested using the CellTiter 96 AQueous One Solution Cell Proliferation Assay (Promega, Madison, WI, USA). Absorbance values were read with a 96-well microplate reader (Synergy HT, Bio-Tek, Winooski, VM, USA) at 490 nm. The percentage (%) of cell viability was calculated by dividing the absorbance of treated cells by the absorbance of the control (untreated) cells.

#### 2.11.5. Measurement of COX-2, IL-1β, IL-2, IL-6, IL-10, and TNF-α

The effect of FPPE on proinflammatory and anti-inflammatory cytokines was evaluated in Raw 264.7 cells. After performing the cellular anti-inflammatory potential assay, Raw 264.7 cells were lysed using 0.5% (*v*/*v*) Triton X-100 for 2 h. After incubation, lysates were mixed with 100 µL of PBS and centrifuged (2000× *g*, 5 min, 4 °C). Supernatants were recovered and stored at −80 °C until use. COX-2 and IL-10 were measured using a Human/Mouse Total COX-2 DuoSet IC and Mouse IL-10 ELISA kits (R&D Systems, Minneapolis, MN, USA) following the manufacturer’s instructions. The absorbance values of cytokines were measured using a Synergy HT plate reader (Bio-Tek Instruments, Inc., Winooski, VT, USA) at 450 nm. Likewise, a MILLIPLEX MAP Mouse Cytokine/Chemokine panel was used to measure IL-1β, IL-2, IL-6, and TNF-α in the supernatant on a Luminex^R^ 200^TM^ System with xPONENT@3.0 software (Luminex, Austin, TX, USA). From the immunoassay, median fluorescent intensity (MFI) data using a polynomial curve-fitting method were used to calculate cytokine concentrations as per the manufacturers’ guidelines.

### 2.12. Statistical Analysis and Data Processing

All results were expressed as the mean ± standard deviation, and all measurements were performed at least in triplicate. Statistical analyses were performed with the JMP Pro 16.0 software (SAS Institute Inc., Cary, NC, USA). One-way and two-way ANOVA were performed for the results obtained and were considered statistically significant at 95% confidence (*p* ≤ 0.05). Differences between treatments were analyzed by Tukey tests and were considered statistically significant at 95% confidence (*p* ≤ 0.05). 

## 3. Results and Discussion

### 3.1. Pineapple Peel Bromatological Analysis and Chemical Composition

The chemical composition of pineapple peels is shown in Table S1. Pineapple peels show a great amount of available carbohydrates (42.29%) and dietary fiber (30.20%). These results are in agreement with previous reports, where the pineapple peel’s chemical composition was 55.52% carbohydrates, 4.39% ashes, and 14.80% crude fiber [24]. 

### 3.2. Solid-State Fermentation of Pineapple Peels with L. plantarum, L. rhamnosus, and A. oryzae

During solid-state fermentation of pineapple peel, the results demonstrate that there were no pH or Aw changes during the fermentation process (Figure S1). It is important to measure these parameters since the growth of microorganisms and the production of enzymes depend on their variation [25,26,27]. 

The optimal pH ranges for the growth of *L. plantarum* and *L. rhamnosus* are between 4 and 9, respectively, while for *A. oryzae*, the optimal pH values fall within the range of 3.8 to 6 [28,29,30]. Lower pH values could suggest variations in the production of phenolic compounds, as well as variances in the production of other bioactive compounds aside from phenolic compounds [27]. Therefore, it is important to monitor these values to ensure that pH values during solid-state fermentation are not getting lower, thereby avoiding the production of non-valuable compounds. 

### 3.3. Total Phenolic Content

Phenolics constitute the most prevalent secondary metabolites in plants, characterized by a structure containing an aromatic ring with at least one hydroxyl substituent [21]. The total phenolic content of pineapple peels treated with solid-state fermentation was investigated. 

**Figure 1:**
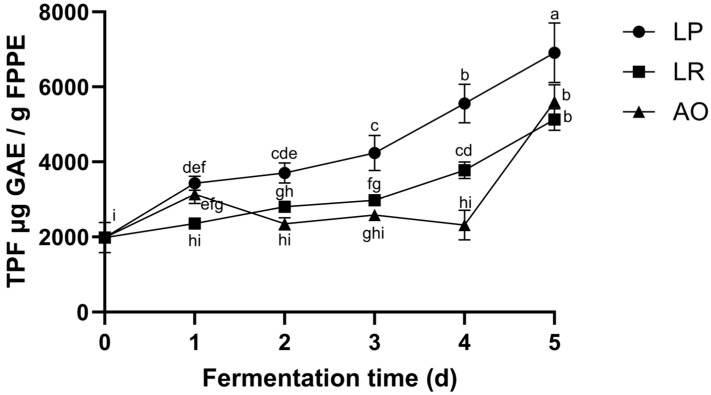
Concentration of total phenolic compounds in pineapple waste fermented for 5 days by *L. rhamnosus* (LR), *L. plantarum* (LP), and *A. oryzae* (AO). Values represent the mean of three replicates with their standard error bars. Different letters among bars indicate statistical differences in the content of total phenolic compounds (TPC) between fermentation times (days) using the Tukey test (*p* < 0.05). Abbreviations: total phenolic compounds (TPC), gallic acid equivalents (GAE), fermented pineapple peel extract (FPPE).

Changes in total phenolic content (TPC) can be observed in Figure 1. The results show a significant increase in the TPC content in pineapple peels after five days of solid-state fermentation across all treatment groups. Notably, *L. plantarum* (LP) exhibits the highest release of TPC, with a significant difference compared to the other treatments (*p* ≤ 0.05), showing a significant increase of 248.11% on the fifth day of fermentation. This was followed by *A. oryzae* (AO) with an 182% increase and *L. rhamnosus* (LR) with a 158.44% increase on the fifth day of fermentation, all compared to the TPC content in pineapple peels on day 0.

As previously reported, phenolic compounds are usually bound to cell wall structural components such as cellulose, hemicellulose, lignin, pectin, and rod-shaped structural proteins [7,31]. The higher release of TPC with *L. plantarum* can be attributed to the enzymes produced during the fermentation processes (e.g., amylase, β-glucosidase, decarboxylase, lactate, dehydrogenase, peptidase, phenolic acid decarboxylase, phenol reductase, proteinase, TanA (tanALp), TanB (tanBLp) esterases). These enzymes can hydrolyze glucosides and break down plant cell walls, liberating phenolic compounds that were initially bound to these plant components [32,33,34]. Conversely, the lower TPC production observed with *L. rhamnosus* and *A. oryzae* may be attributed to the distinct enzymes generated during their respective fermentation processes [32,34]. 

Regarding *A. oryzae*, previous studies have indicated that it secretes enzymes like α-amylase, β-glucosidase, and cellulase during solid-state fermentation, contributing to the release of bound phenolic compounds [18,35,36]. However, enzyme production by *A. oryzae* typically begins to rise between the fourth and fifth days of fermentation, as was reported in previous studies [37,38,39]. This timing agrees with the observed increase in phenolic compound production in the current study.

### 3.4. HPLC-DAD and LC/MS-TOF Bioactive Compounds Quantification and Characterization

The profile of bioactive compounds obtained from the fermented pineapple peel extracts (FPPE) in *L. rhamnosus*, *L. plantarum,* and *A. oryzae* FPPE is shown in Table 1. Thirty compounds were identified in both the control and treated samples. The phenolic profile of pineapple peel agrees with previous reports [20,40]. However, the concentration of identified individual phenolics varied with the effect of the applied treatment and fermentation time (Table 2).

Changes in individual bioactive compound content can be observed in Table 2. Ten of these compounds were only detected in the control sample. 

Fermentation with *L. rhamnosus* led to an immediate and significant increase (on day 1) in the content of S-Sinapyl-L-cysteine (63.2%) and feruloyl hexoside (56.4%) compared to the control. Conversely, *L. plantarum* induced an increase in *p*-Coumaroyl-feruloyl glycerol (935.1%), S-Sinapyl-L-cysteine (83.6%), and N-L-γ-Glutamyl-S-p-coumaryl-L-cysteine (164.9%).

In general, the maximum accumulation of phenolics was observed in *L. plantarum* FPPE after 5 d of fermentation. The four main compounds overproduced by this microorganism were S-coniferyl-L-cysteine, sinapic acid, coumaric acid derivative, and 4-hydroxy-2,5-dimethyl-3(2H)-furanone (furaneol), accounting for 22.67%, 15.24%, 11.43%, and 5.57% of the TPC of FPPE, respectively.

Regarding *L. rhamnosus* FPPE, the characterization of phenolic compounds shows that the most abundant compounds were ferulic acid, feruloyl hexoside, S-coniferyl-L-cysteine, gallic acid glucoside, and 4-hydroxy-2,5-dimethyl-3(2H)-furanone (furaneol). On the 5th fermentation day, ferulic acid emerged as the primary phenolic compound, accounting for 27.57% of the TPC of FPPE, followed by feruloyl hexoside (6.95%) and S-coniferyl-L-cysteine (6.33%).

In the case of *A. oryzae* FPPE, the characterization of TPC shows that the most abundant compounds were 4-methoxy-2,5-dimethyl-3(2H)-furanone, ferulic acid, *p*-coumaric acid derivative, and sinapic acid. On the 5th fermentation day, 4-methoxy-2,5-dimethyl-3(2H)-furanone (Furaneol) was the main phenolic compound, constituting 39.26% of the TPC of FPPE, followed by an unidentified compound (12.20%) and ferulic acid (8.92%).

These studies demonstrate the presence of phenolic acids after enzymatic β-glucosidase hydrolysis, suggesting that the hexosides initially present in pineapple peel are transformed into phenolic acids through the action of this enzyme. This observation supports the notion that the microorganisms employed in the solid-state fermentation of pineapple peel in this study produce the same enzyme, thereby explaining the variations in the profiles of phenolic compounds when compared to the FPPE profiles at day zero of fermentation.

The most prevalent compounds derived from the solid-state fermentation of pineapple peels with *L. rhamnosus*, *L. plantarum*, and *A. oryzae* hold significant potential for antioxidant applications in the food and cosmetic industries. Particularly, 4-Methoxy-2,5-dimethyl-3(2H)-furanone (Furaneol) has been previously employed as a flavoring agent, owing to its sensory attributes [41,42,43].

### 3.5. 2,2-Diphenyl-1-Picrylhydrazyl (DPPH) Scavenging Activity

**Figure 2:**
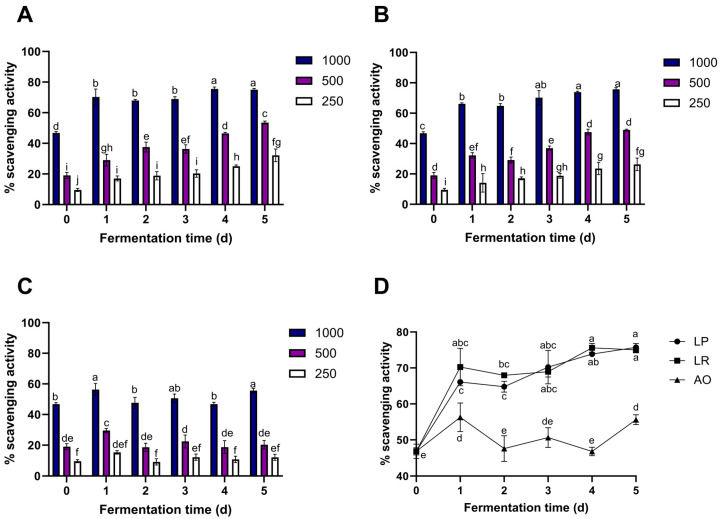
2,2-Diphenyl-1-picrylhydrazyl (DPPH) scavenging activity of fermented pineapple peel extract (250, 500, and 1000 µg/mL) by (**A**) *L. rhamnosus* (LR), (**B**) *L. plantarum* (LP), and (**C**) *A. oryzae* (AO). (**D**) DPPH scavenging activity (%) at 1000 µg/mL FPPE of each microorganism. Values represent the mean of three replicates with their standard error bars. ^a–j^ Different letters among the bars indicate statistical differences in the DPPH scavenging activity of each FPPE between fermentation times (days) using the Tukey test (*p* < 0.05). Abbreviations: 2,2-diphenyl-1-picrylhydrazyl (DPPH), fermented pineapple peel extract (FPPE).

As it is known, many biologically active molecules in plants may contribute to antioxidant capacities, and pineapple is not an exception. The antioxidant activity of FPPE was determined in terms of the proportion (%) of DPPH scavenged, and values are shown in Figure 2. 

In the case of *L. rhamnosus* (Figure 2A), a significant increase in scavenging activity was observed in *L. rhamnosus* FPPE in a time-dose-dependent manner when using concentrations of 250 and 500 µg/mL. Conversely, at a concentration of 1000 µg/mL of *L. rhamnosus* FPPE, scavenging activity exhibits a significant increase (50.05%) after just one day of fermentation compared to the control (*p* ≤ 0.05), and this enhanced activity was maintained without significant differences until day 5. 

Furthermore, the highest induced antioxidant activity with *L. rhamnosus* treatment was observed after 4 d of fermentation. It was observed that a significant increase of 238.52%, 181.73%, and 62.44% was achieved with concentrations of 250 µg/mL, 500 µg/mL, and 1000 µg/mL FPPE, respectively, in comparison to their respective control groups. 

Regarding *L. plantarum*, the scavenging activity (%) of FPPE also displays a time-dependent increase (Figure 2B). At a concentration of 250 µg/mL of *L. plantarum* FPPE, scavenging activity exhibits a significant increment (48.52%) after one day of fermentation compared to the control (*p* ≤ 0.05). However, this heightened activity was consistently sustained on days 2, 3, and 4, with no significant variations. On the other hand, concentrations of 500 µg/mL and 1000 µg/mL FPPE show a significant daily increase in scavenging activity (%), reaching the highest level after 5 d of fermentation (155.74% and 61.65%, respectively). 

Nevertheless, the fermentation treatments did not induce an immediate increment in the radical scavenging activity of pineapple peels (Figure 2C). 

When the interaction between microorganisms and fermentation times at 1000 µg/mL is compared (Figure 2D), results show that the radical scavenging activities of pineapple peel extracts fermented by *L. plantarum* and *L. rhamnosus* were not statistically different on days 3, 4, and 5 (*p* ≤ 0.05). Moreover, *L. plantarum* and *L. rhamnosus* FPPE at 1000 µg/mL concentrations had better DPPH scavenging activity compared to *A. oryzae* FPPE at day 5 at the same concentration (57.51% and 56.02%, respectively). 

The results obtained from antioxidant activity assays of FPPE correlate with the increase in TPC of FPPE over time. These findings are consistent with prior studies evaluating the antioxidant activity of various pineapple residue extracts using the DPPH scavenging activity assay. For instance, Othman et al. [44] reported a 26.24% DPPH scavenging activity at a 500 µg/mL concentration of pineapple peel extract. In addition, de Oliveira et al. [45] reported an increase in DPPH activity on the concentration of pineapple residue extract (a mix of pineapple pulp, seeds, and peels), achieving 20% inhibition at a concentration of 100 µg/mL. Moreover, Hossain and Rahman [46] reported a high DPPH scavenging activity of 84.3% at a concentration of 100 µg/mL for methanolic pineapple extract (from ripe pineapple pulp). Afsharnezhad et al. [47] also reported a DPPH scavenging activity of 52.32% with a methanolic pineapple peel extract, which is similar to the result obtained from non-fermented pineapple peel (46.83%) at day 0. 

Variations in antioxidant activity can primarily be ascribed to disparities in the phenolic content within the extracts. It has been well documented that the antioxidant capacity of phenolic compounds primarily stems from the presence of methoxy, hydroxyl, and carboxylic acid groups. These functional groups play a pivotal role in neutralizing free radicals, quenching singlet and triplet oxygen, and decomposing peroxides, which can play an important role in neutralizing free radicals, quenching singlet and triplet oxygen, or decomposing peroxides [8]. 

### 3.6. Lipid Peroxidation Inhibition

Lipid peroxidation occurs when there is a large amount of reactive oxygen species (ROS), which can induce a cascade of reactions that cause oxidative stress in cell membranes, creating lipid radicals that can potentially damage proteins and DNA. Lipid peroxidation affects membrane fluidity, damages membrane proteins, and deactivates membrane receptors [48]. 

**Figure 3:**
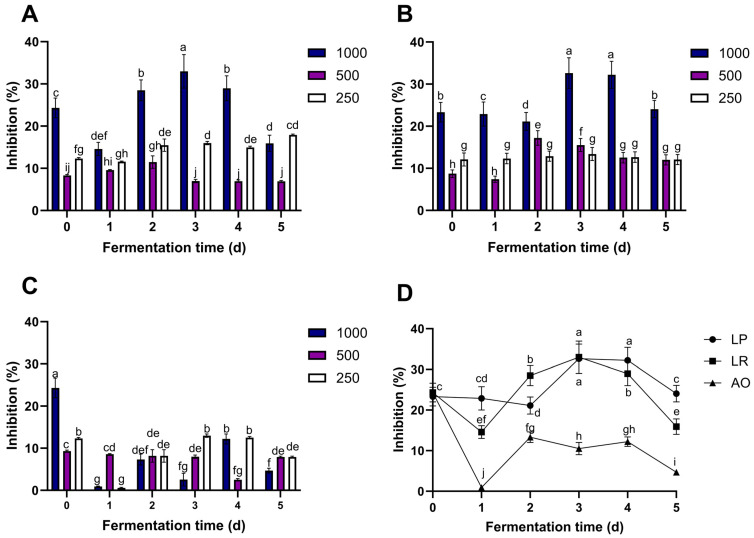
Lipid peroxidation inhibition of fermented pineapple peel extract (250, 500, and 1000 µg/mL) by (**A**) *L. rhamnosus* (LR), (**B**) *L. plantarum* (LP)*,* and (**C**) *A. oryzae* (OA). (**D**) Lipid peroxidation inhibition of 1000 µg/mL extracts of each microorganism. Values represent the mean of three replicates with their standard error bars. Different letters among bars indicate statistical differences in the lipid peroxidation inhibition of each FPPE between fermentation times (days) using the Tukey test (*p* < 0.05). Abbreviation: fermented pineapple peel extract (FPPE).

As shown in Figure 3, the results indicate that FPPE from *L. rhamnosus*, *L. plantarum,* and *A. oryzae* exhibits potential in mitigating lipid peroxidation (Figure 3A). For *L. rhamnosus*, it was observed that concentrations of 250 µg/mL and 500 µg/mL FPPE did not exert a significant effect on lipid peroxidation modulation. Conversely, a concentration of 1000 µg/mL consistently and significantly increased lipid peroxidation inhibition after 2 days of fermentation. 

In the case of *L. plantarum* (Figure 3B), the highest lipid peroxidation inhibition (%) was at day 3 for *L. rhamnosus* (35.51%) and at days 3 and 4 for *L. plantarum* (33.99% and 32.38%, respectively) with 1000 µg/mL of FPPE.

Conversely, when considering lipid peroxidation inhibition with *A. oryzae* FPPE (Figure 3C), the peak inhibition was achieved on day 0 of fermentation at all three concentrations. Subsequently, there was a significant decrease in the percentage of inhibition. The higher decrease was observed from day 0 to day 1 with 250 µg/mL (−363.31%) and 1000 µg/mL (−96.21%) of FPPE, which could be due to the initial liberation of bioactive compounds bound in pineapple peel.

When the interaction between microorganisms and fermentation time at 1000 µg/mL is compared (Figure 3D), results show that lipid peroxidation inhibition of pineapple peel extracts fermented by *L. plantarum* and *L. rhamnosus* was not statistically different on days 3 and 4 (*p* ≤ 0.05). Moreover, *L. plantarum* and *L. rhamnosus* FPPE at 1000 µg/mL concentrations had better lipid peroxidation inhibition compared to *A. oryzae* FPPE during all the fermentation processes. 

The results align with several studies that have evaluated the lipid peroxidation inhibition (%) of pineapple extracts. For instance, De Oliveira et al. [45] demonstrated the impact of a pineapple by-product extract, consisting of both pulp and peel components, at a concentration of 500 µg/mL. This extract exhibits a significant capacity to inhibit lipid peroxidation, with a 20% reduction observed after 30 min of reaction. Moreover, in vivo studies involving rats subjected to alcohol-induced oxidative stress further highlighted the efficacy of pineapple peel extract in mitigating lipid peroxidation. Treatment with pineapple peel extract at a dosage of 2.5 mL/kg bw (body weight) resulted in a remarkable 60.16% reduction in malondialdehyde (MDA) levels—an important biomarker of lipid peroxidation [49]. Similarly, another in vivo study focused on the impact of pineapple peel extract on total phospholipids and lipid peroxidation in rat brain tissues and demonstrated that treatment with pineapple peel extract at 2.5 mL/kg bw led to a significant (72.50%) reduction in MDA levels [50]. 

As demonstrated in this experiment, solid-state fermentation processes have the potential to release compounds responsible for inhibiting lipid peroxidation. Variations in lipid peroxidation inhibition (%) between FPPEs derived from different microorganisms and fermentation days may be attributed to the liberation of distinct phenolic compounds during each fermentation period. 

Subsequent assays did not involve the assessment of *A. oryzae* FPPE due to its low performance, characterized by low DPPH scavenging activity (%) and limited inhibition of lipid peroxidation (%), as observed in the preliminary assays. Consequently, the subsequent experiments exclusively employed *L. rhamnosus* and *L. plantarum* FPPE to investigate cellular antioxidant capacity, cellular anti-inflammatory potential, COX-2 production, IL-10 production, TNF-α production, IL-1β production, and IL-6 production.

### 3.7. Cellular Antioxidant Capacity

**Figure 4:**
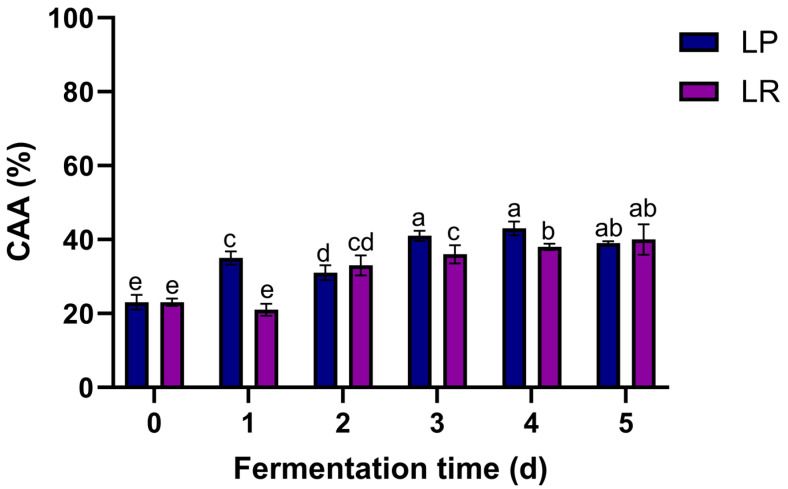
Cellular antioxidant capacity of fermented pineapple peel extracts (25 µg/mL) by *L. rhamnosus* (LR) and *L. plantarum* (LP) in Caco2 cells. Values represent the mean of three replicates with their standard error bars. Different letters among bars indicate statistical differences in the cellular antioxidant capacity between microorganisms and fermentation time (days) using the Tukey test (*p* < 0.05).

The cellular antioxidant capacity assay is a valuable tool for measuring the antioxidant activity of bioactive compounds in different cell cultures [51,52]. The cellular antioxidant activity of fermented pineapple peel extract (25 µg/mL) by *L. rhamnosus* and *L. plantarum* in Caco2 cells is shown in Figure 4.

While cellular antioxidant activity did not show significant differences between microorganisms during fermentation with *L. rhamnosus* and *L. plantarum*, the duration of the fermentation process positively influenced cellular antioxidant activity. There was a significant and constant increase in cellular antioxidant activity observed with FPPE from day 2 to day 5 with *L. rhamnosus* and *L. plantarum* when compared to day 0.

The highest cellular antioxidant capacity was achieved with FPPE after 5 days of fermentation with both microorganisms, resulting in a statistically significant increase (*p* ≤ 0.05) of 73.91% and 69.56% with *L. rhamnosus* and *L. plantarum*, respectively, compared to day 0.

The results obtained from the cellular antioxidant activity assay of FPPE show similar behavior to the DPPH scavenging activity assay. Since fermentation can transform phenolic compounds into simpler and smaller molecules, their permeability to the cell is enhanced [53]. The cellular antioxidant capacity of FPPE in later days can be correlated to the amount of ferulic acid that is liberated after the solid-state fermentation of pineapple peels [54,55]. 

In agreement with these results, the influence of ferulic acid on cellular antioxidant activity has been previously reported. For example, Gaxiola-Cuevas et al. [56] demonstrated that a phenolic compound extract from maize tortillas, mainly composed of ferulic acid, had a cellular antioxidant capacity of 72.8% to 77.5% compared to untreated cells. Moreover, another study revealed that ferulic acid obtained from the solid-state fermentation process of wheat bran had a cellular antioxidant capacity of 63.63% compared to AAPH-induced non-treated cells. This may be explained because ferulic acid inhibits free radical-generating enzymes and improves cell protective activity [57].

### 3.8. Cellular Anti-Inflammatory Potential

The effects of fermented pineapple peel extract (25 µg/mL) by *L. rhamnosus* and *L. plantarum* on nitric oxide (NO) production and COX-2 protein expression in activated macrophages were tested.

**Figure 5:**
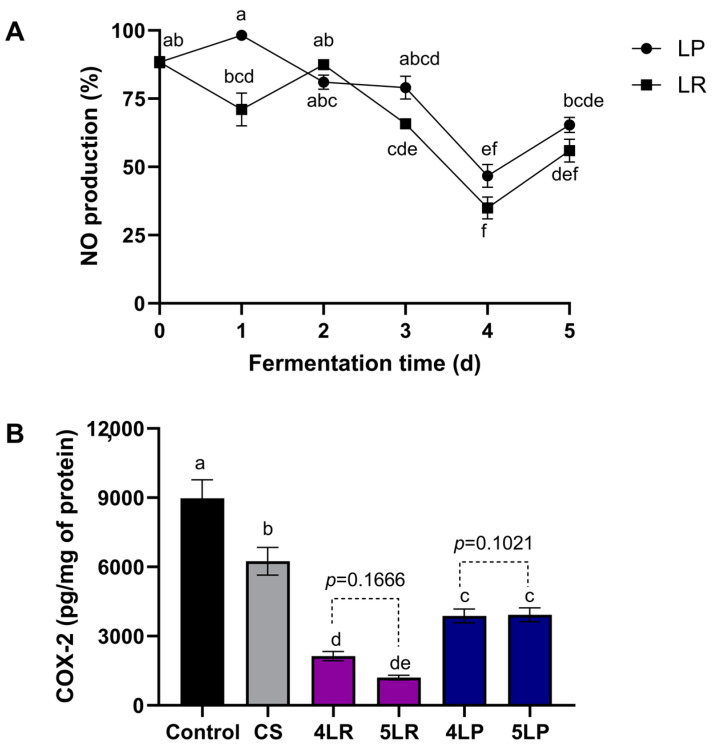
Effect of *L. rhamnosus* (LR) and *L. plantarum* (LP) fermented pineapple peel extracts on (**A**) nitric oxide as an inflammatory biomarker and (**B**) COX-2 production by macrophage Raw 264.7 cells stimulated with 1 μg/mL lipopolysaccharide. Bars represent the means of three replicates ± standard error. Different letters indicate a statistically significant difference between all treatments, as determined by Tukey’s HSD test (*p* < 0.05). Abbreviations: NO: nitric oxide; CS: FPPE on day 0; 4LR: *L. rhamnosus* FPPE on day 4; 5LR: *L. rhamnosus* FPPE on day 5; 4LP: *L. plantarum* FPPE on day 4; 5LP: *L. plantarum* FPPE on day 5; FPPE: fermented pineapple peel extracts.

As seen in Figure 5A, Raw 264.7 cells show a significant decrease in NO production in pineapple peel extracts from day 3 of fermentation by *L. rhamnosus* (25.5%) and day 4 by *L. plantarum* (47.1%), compared to day 0, respectively. 

Although fermentation was not significantly different between microorganisms, the fermentation process positively affected nitric oxide production. The treatments that had the lowest nitric oxide production compared to FPPE at day 0 (88.36%) were *L. rhamnosus* (38%) and *L. plantarum* (46.7%) FPPE at day 4 (*p* ≤ 0.05). 

It was observed that there was a significant increase in NO production with *L. rhamnosus* (59.9%) and *L. plantarum* (40.0%) FPPE at day 5 (*p* ≤ 0.05) compared to day 4. This increase can be attributed to the higher concentration of phenolic compounds, including ferulic acid, which has been associated with pro-inflammatory activity at elevated concentrations [58,59,60]. The enzymes produced by *L. rhamnosus* and *L. plantarum* during fermentation hydrolyze ferulic acid glyceride bonds, resulting in the availability of phenolic acids that enhance their anti-inflammatory properties [53].

A study by Pongjanta and Chansiw [61] showed that their non-fermented pineapple peel extract, derived from two pineapple varieties from Thailand, exhibited NO production levels of 77.28%, 71.81%, and 64.47% for the Nanglae variety and 66.73%, 65.35%, and 63.67% for the Phulae variety at extract concentrations of 10 µg/mL, 100 µg/mL, and 1000 µg/mL, respectively. NO production in this study shows slight changes even at higher concentrations (1000 µg/mL). Differences between these findings and previous reports could be attributed to variations in plant variety and maturity stage.

The effects of FPPE on COX-2 protein expression were detected by ELISA analysis. Results demonstrate that *L. rhamnosus* and *L. plantarum* FPPE at 25 µg/mL concentrations reduced the production of COX-2 (>59.07%) (Figure 5B).

The treatment that had the highest reduction (85.79%) in COX-2 production (1290 pg/mg protein) compared to the untreated control (9081.15 pg/mg protein) was *L. rhamnosus* FPPE at day 5 of fermentation (5LR) (*p* ≤ 0.05), followed by day 4 of fermentation (2379.61 pg/mg protein) with the same microorganism (4LR) (*p* ≤ 0.05).

Moreover, when comparing COX-2 production between 4LR and 5LR, there were no statistically significant differences between these two treatments (*p* ≤ 0.05). Likewise, there was no statistically significant difference between treatments with *L. plantarum* FPPE on day 4 (3555.44 pg/mg protein) and day 5 (3717 pg/mg protein) (*p* ≤ 0.05). 

FPPE at day 0 (CS) shows no significant difference in the production of COX-2 (6617.33 pg/mg protein) compared to the untreated control. This aligns with findings from Moraes et al. [62], who evaluated COX-2 inhibition in LPS-induced Raw 264.7 cells using pineapple extracts at a concentration of 50 µg/mL. These results suggest that solid-state fermentation releases anti-inflammatory compounds capable of inhibiting COX-2 production.

A greater reduction in the production of COX-2 could be partly attributed to the higher ferulic acid content in *L. rhamnosus* FPPE on day 5. Mir et al. [63] demonstrated that ferulic acid can protect against LPS-induced acute kidney injury in Balb/c mice, achieving COX-2 inhibition with concentrations of 100 mg/kg and 50 mg/kg.

In another study, the inhibitory potential of *Terminalia bellirica* (Gaertn) Roxb fruit extracts was assessed in LPS-induced Raw 264.7 cells, showing a significant COX-2 inhibition attributed to its phenolic compound composition. Notably, one of the main compounds of this fruit was ferulic acid [64]. Furthermore, Villela-Castrejón et al. [53] demonstrated that spray-dried nejayote, rich in ferulic acid (constituting 77.8% of the polyphenols) after in vitro digestion, exhibited an inhibitory effect on COX-2 in LPS-induced Raw 264.7 cells.

These results indicate that the inhibitory effect of FPPE on NO production might be responsible for suppressing COX-2 protein expression production in cells stimulated with LPS. Additionally, the observed reduction in anti-inflammatory cytokines may be attributed to the ability of the present phenolic compounds to inhibit nuclear factor-ĸB (NF-ĸB), a pivotal transcription factor responsible for the activation of genes related to inflammatory mediators, including inducible nitric oxide synthase (iNOS) and cyclooxygenases (COXs) [23]. 

### 3.9. Cytokines Production 

**Figure 6:**
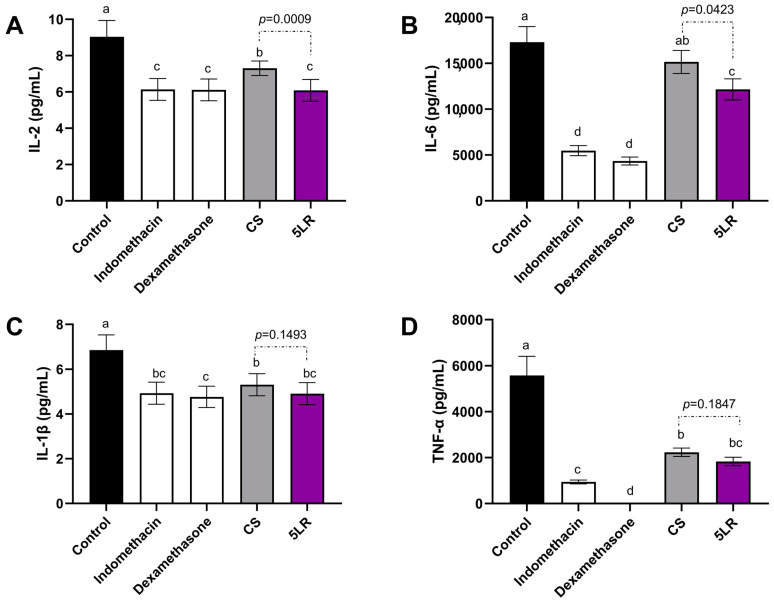
Effect of FPPE at day 0 (CS) and *L. rhamnosus* at day 5 (5LR) fermented pineapple peel extracts on the production of inflammatory cytokines by macrophage Raw 264.7 cells stimulated with 1 μg/mL lipopolysaccharide: (**A**) IL-2, (**B**) IL-6, (**C**) IL-1β, and (**D**) TNF-α. Bars represent the means of three replicates ± standard error. Different letters indicate a statistically significant difference between all treatments, as determined by Tukey’s HSD test (*p* < 0.05). Abbreviations: FPPE: fermented pineapple peel extracts; CS: FPPE on day 0; 5LR: *L. rhamnosus* FPPE on day 5.

Because inflammation is a complex system where cytokines are involved, it is important to measure their activity. The influence of pineapple peel extracts fermented by *L. rhamnosus* and *L. plantarum* on the release of anti-inflammatory cytokine IL-10 (interleukin 10), and pro-inflammatory cytokines IL-6 (interleukin 6), IL-2 (interleukin 2), IL-1β (interleukin 1β), and TNF-α (tumor necrosis factor-alpha) was evaluated. The results are shown in Figure 6.

**Figure 7:**
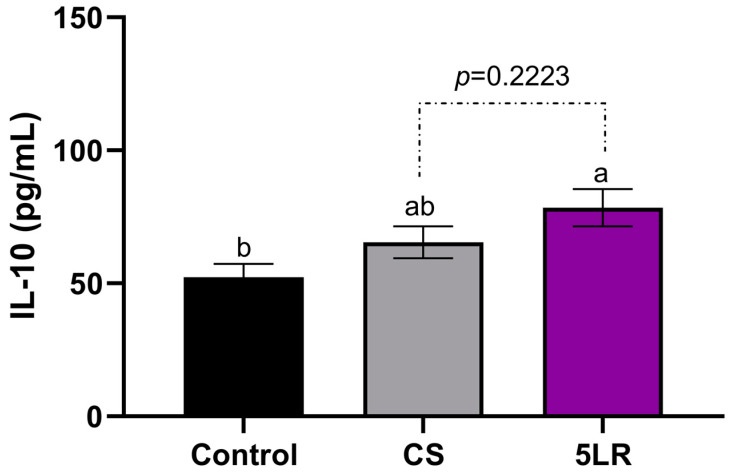
Effect of FPPE at day 0 (CS) and *L. rhamnosus* at day 5 (5LR) fermented pineapple peel extracts on the production of the anti-inflammatory cytokine IL-10 by macrophage Raw 264.7 cells stimulated with 1 μg/mL lipopolysaccharide. Bars represent the means of three replicates ± standard error. Different letters indicate a statistically significant difference between all treatments, as determined by Tukey’s HSD test (*p* < 0.05). Abbreviations: FPPE: fermented pineapple peel extracts; CS: FPPE on day 0; 5LR: *L. rhamnosus* FPPE on day 5.

*L. rhamnosus* FPPE at day 5 (5LR) reduced the production of IL-2, IL-6, IL-1β, and TNF-α (Figure 6) and increased the production of IL-10 in LPS-treated Raw 264.7 cells (Figure 7). There was a statistically significant difference between all the treatments compared to the untreated control (*p* ≤ 0.05).

Treatments with 5LR and CS show a significant decrease in IL-2 production by 32.61% and 26.67%, compared to control, respectively. A statistically significant difference was observed between these two treatments (*p* = 0.0009). Furthermore, *L. rhamnosus* FPPE on day 5 (5LR) exhibits a similar effect to that of Indomethacin (1 µg/mL) and Dexamethasone (1 µg/mL), two reference anti-inflammatory agents, in reducing IL-2 production (32.06% and 34.45%, respectively).

A significant reduction in IL-6 production was observed with the 5LR treatment (−22.78%) compared to the untreated control. However, both Indomethacin (−60.56%) and Dexamethasone (−68.95%) demonstrate a higher reduction compared to the control. Notably, the CS treatment exhibits no statistically significant difference when compared to the untreated control (*p* ≤ 0.05) (Figure 6B). These findings align with those reported by Ajayi et al. [65] who noted a 27.27% reduction in IL-6 production in rats with high-fat diet-induced memory impairment and anxiety-like behavior after receiving a pineapple peel extract (200 mg/kg) compared to untreated rats.

A significant decrease of 39.6% in IL-1β production (Figure 6C) was observed with *L. rhamnosus* FPPE on day 5 (5LR) compared to the untreated control (*p* ≤ 0.05). No statistically significant differences were observed between treatments. 

In addition, treatments with 5LR and CS show a significant decrease in TNF-α production (Figure 6D) by 67.34% and 60.22%, compared to control, respectively. 

On the other hand, there was a significant increase of 31.8% in IL-10 production with the 5LR treatment when compared to the untreated control (Figure 7). Villela-Castrejón et al. [53] demonstrated that spray-dried nejayote (where ferulic acid was 77.8% of TPC) at 100 µg/mL concentration had an important effect on the production of IL-10 after 2, 8, and 12 h of absorption; thus, it decreased afterward. This can be related to the fact that phenolic compounds at higher concentrations have pro-inflammatory activity [58,59,60]. 

Studies on the reduction of the production of IL-2, IL-6, IL-1β, and TNF-α with ferulic acid have been reported. For example, Hu et al. [66] demonstrated that a basal diet containing 4000 mg/kg of ferulic acid reduced the serum levels of IL-2, IL-6, IL-1β, and TNF-α (≈57.4%, ≈15.56%, ≈31.43%, and ≈50%, respectively) in lipopolysaccharide-challenged piglets. As well as a study by Seenak et al. [67] in which they reported a decrease in IL-6 (32.83%) and IL-1β (34.40%) and no statistical difference in TNF-α production on cardiac inflammation in high cholesterol diet-fed rats with pineapple powder (200 mg/kg/day). Moreover, Ma et al. [68] demonstrated that a phenolic extract of black chokeberry reduced the production of COX-2 (25%), TNF-α (14.28%), IL-6 (33.33%), and IL-1β (50%) in LPS-treated RAW 264.7 cells and increased the production of the anti-inflammatory IL-10 (33.33%) cytokine. In addition, Kim et al. [69] evaluated the anti-inflammatory effects of different rich-phenolic compounds in peanut skin extracts on the production of inflammatory TNF-α, IL-6, and IL-1β cytokines in LPS-induced RAW 264.7 cells. Results demonstrate that these extracts reduced the production of these inflammatory cytokines by 33.33%, 75%, and 25%, respectively.

**Figure 8:**
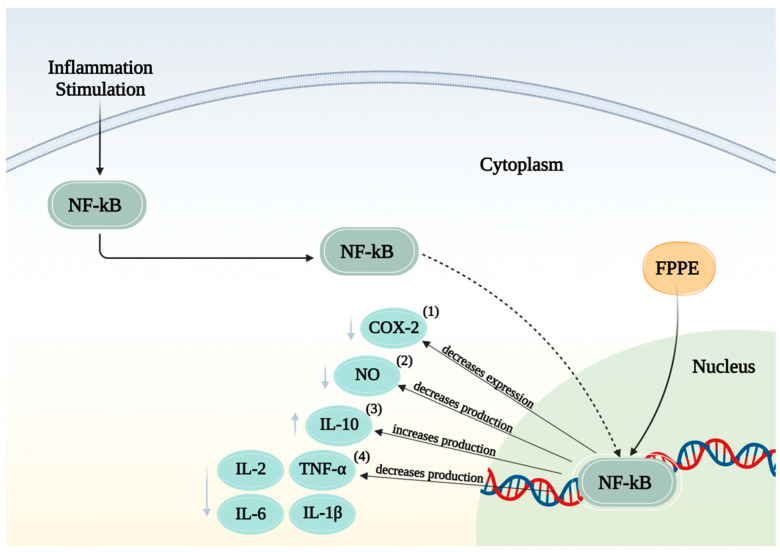
Effects of fermented pineapple peel extracts (FPPE) on inflammation stimulation.

The proposed mechanism of action of FPPE for its potential anti-inflammatory effects is shown in Figure 8. 

Activation of the NF-κB pathway leads to the activation of NFκB dimers, which in turn initiate the transcription of genes encoding various inflammation-related proteins involved in inflammation, including cyclooxygenase (COX-2), inducible nitric oxide synthase (iNOS), interleukin 10 (IL-10), interleukin 2 (IL-2), interleukin 6 (IL-6), interleukin 1β (IL-1β), and tumor necrosis factor-alpha (TNF-α). The findings from this study indicate that FPPE resulted in a reduction in the expression of COX-2 (1) and NO (2), along with an increase in the expression of IL-10 (3). Additionally, there was a decrease in the expression of IL-2, IL-6, IL-1β, and TNF-α (4). This figure was created using Biorender.com. Accessed on 23 October 2023.

## 4. Conclusions

This study demonstrates that solid-state fermentation of pineapple peels using lactic acid bacteria (*L. plantarum* and *L. rhamnosus*) and filamentous fungi (*A. oryzae*) leads to an increased release of phenolic compounds that are typically bound within the pineapple peel matrix. Furthermore, this enhanced release of phenolic compounds also increased their potential as antioxidants and anti-inflammatory agents. The anti-inflammatory activity was correlated to the significant inhibition of pro-inflammatory cytokines, including COX-2, IL-2, IL-6, IL-1β, and TNF-α, and the promotion of IL-10 production, which is known for its potent anti-inflammatory properties.

Remarkably, the profiles of phenolic compounds from pineapple peel were found to vary depending on the microorganism used for solid-state fermentation. Consequently, different microorganisms can be selected based on the intended application. For instance, for higher total phenolic compound (TPC) production, solid-state fermentation with *L. plantarum* over 5 days is recommended. Conversely, for greater nitric oxide (NO) inhibition, solid-state fermentation with *L. rhamnosus* for 4 days is more suitable. Likewise, *L. plantarum* and *L. rhamnosus* produced a significant amount of ferulic acid and vanillin, compounds commonly used in the food and cosmetic industries. In contrast, *A. oryzae* generates furanones, which find widespread application in the food industry, imparting specific aromas to food products such as the pineapple scent. 

Since a great variety of value-added compounds can be produced using pineapple waste, it is essential to thoroughly analyze its production yields and scalability in the industrial field. 

## Figures and Tables

**Table 1:** Identification of bioactive compounds obtained from the fermented pineapple peel extracts (FPPE).

Peak N°	RT (min)	Compound	λ Max (nm)	*m*/*z* [M + H]^+^	Molecular Formula	CAS Number
1	7.77	4-Methoxy-2,5-dimethyl-3(2H)-furanone	270	143	C_7_H_10_O_3_	4077-47-8
2	8.58	L-Tyrosine	270	182	C_9_H_11_NO_3_	60-18-4
3	10.39	Serotonin	276, 296	177	C_10_H_12_N_2_O	50-67-9
4	12.47	Caffeoyl Isocitrate	301	377 (Na + adduct)	C_15_H_11_O_10_^-3^	25245208 ^1^
5	12.79	Gallic acid	272	171	C_7_H_6_O_5_	149-91-7
6	16.42	4-Hydroxy-2,5-dimethyl-3(2H)-furanone-hexoside	277	291	C_12_H_18_O_8_	121063-56-7 ^2^
7	17.68	Caffeoyl feruloyl spermidine	242, 297, 327	484	C_26_H_33_N_3_O_6_	129821576 ^1^
8	18.72	4-Hydroxy-2,5-dimethyl-3(2H)-furanone (Furaneol)	287	129	C_6_H_8_O_3_	3658-77-3
9	21.28	Syringyl hexoside	278, 299	383 (Na + adduct)	C_15_H_20_O_10_	129719980 ^3^
10	22.09	N-L-γ-Glutamyl-S-*p*-coumaryl-L-cysteine	278	383	C_17_H_22_N_2_O_6_S	129850043 ^1^
11	22.57	L-Tryptophan	279	205	C_11_H_12_N_2_O_2_	73-22-3
12	24.12	3,4-Dimethoxyphenyl βD-glucoside	278	317	C_14_H_20_O_8_	10313649 ^1^
13	25.24	Gallic acid glucoside	280	333	C_13_H_16_O_10_	10088114 ^1^
14	26.50	4-Hydroxy-2,5-dimethyl-3(2H)-furanone-malonyl hexoside	277	377	C_15_H_20_O_11_	131750900 ^1^
15	27.17	N.I.	270	373	----	-------
16	29.67	Sinapoyl hexoside	330	409 (Na + adduct)	C_17_H_22_O_10_	13787030 ^1^
17	29.88	S-Coniferyl-L-cysteine	269, 303	284	C_13_H_17_NO_4_S	129850384 ^1^
18	30.82	N.I.	324, 290, 240	393	----	-------
19	31.54	S-Sinapyl-L-cysteine	278	314	C_14_H_19_NO_5_S	15553258 ^1^
20	33.54	*p*-Coumaroyl-feruloyl glycerol	243, 331, 300	415	C_22_H_22_O_8_	14135372 ^1^
21	36.94	S-Coniferyl Glutathione	269, 300	470	C_20_H_27_N_3_O_8_S	72721517 ^1^
22	37.49	Coumaric acid derivative	308	367	----	-------
23	37.57	S-Sinapyl Glutathione	278	500	C_21_H_29_N_3_O_9_S	72720606 ^1^
24	38.94	N-L-γ-Glutamyl-S-coniferyl-Cysteine	316	413	C_18_H_24_N_2_O_7_S	72721515 ^1^
25	39.56	N-L-γ-Glutamyl-S-sinapyl-L-Cysteine	277	443	C_19_H_26_N_2_O_8_S	15553259 ^1^
26	39.70	Ferulic acid	322, 292, 236	195	C_10_H_10_O_4_	537-98-4
27	40.15	Sinapic acid	234, 322	225	C_11_H_12_O_5_	7362-37-0
28	40.29	(di-E,E)-N,N′-Di Feruloyl Spermidine	242, 329	498	C_27_H_35_N_3_O_6_	29664291 ^1^
29	41.44	N.I.	275	631	----	-------
30	41,441	N.I.	275	631		
30	41.53	Feruloyl hexoside	237, 292, 320	357	C_16_H_20_O_9_	7196-71-6 ^4^

N.I.: Not identified; CAS: Chemical Abstracts Service; ^1^ PubChem CID; ^2^ Tentatively identified as furaneol 4-glucoside; ^3^ Tentatively identified as Syringoyl-glucoside; ^4^ Tentatively identified as 1-O-Feruloylglucose.

**Table 2:** Concentration of individual bioactive compounds obtained from the fermented pineapple peel extracts (FPPE) treated with *L. rhamnosus* (LR), *L. plantarum* (LP), *and A. oryzae* (AO) for 5 days.

		**Bioactive Compound Concentration (mg/g DW) ^1,2,3^**													
**Treatment**	**Fermentation Time (d)**	**4-Methoxy-2,5-dimethyl-3(2H)-furanone**		**L-Tyrosine**		**Caffeoyl Isocitrate**		**Gallic Acid**		**4-Hydroxy-2,5-dimethyl-3(2H)-furanone-hexoside**		**Caffeoyl Feruloyl Spermidine**		**4-Hydroxy-2,5-dimethyl-3(2H)-furanone**	
**Control**	**0**	ND	c	35.00 ± 4.08	e	ND	f	ND	d	65.00 ± 2.31	e	ND	c	ND	f
**LR**	**1**	ND	c	50.00 ± 0.10	d	10.00 ± 0.32	e	15.00 ± 3.53	c	143.33 ± 3.33	a	20.00 ± 0.10	b	56.67 ± 3.33	e
	**2**	ND	c	55.00 ± 5.00	d	30.00 ± 0.10	de	43.33 ± 6.67	bc	126.67 ± 3.33	b	20.00 ± 0.45	b	86.67 ± 8.82	e
	**3**	ND	c	50.00 ± 0.30	bc	85.00 ± 5.00	b	50.00 ± 2.50	b	100.00 ± 5.77	c	20.00 ± 0.33	b	90.00 ± 10.00	d
	**4**	ND	c	66.67 ± 3.33	b	135.00 ± 15.00	a	60.00 ± 10.00	b	96.67 ± 6.67	c	33.33 ± 3.30	b	150.00 ± 10.00	c
	**5**	ND	c	76.67 ± 3.33	a	96.67 ± 3.33	b	70.40 ± 5.77	a	66.67 ± 3.33	e	90.00 ± 7.32	a	203.33 ± 8.82	b
**LP**	**1**	ND	c	50.00 ± 0.14	d	10.00 ± 0.32	e	ND	d	153.33 ± 3.33	a	ND	c	56.67 ± 8.82	e
	**2**	ND	c	50.00 ± 0.43	d	33.33 ± 8.92	d	ND	d	123.33 ± 3.33	b	ND	c	100.00 ± 10.00	d
	**3**	ND	c	63.33 ± 3.33	cd	56.67 ± 3.33	c	ND	d	96.67 ± 3.33	c	ND	c	130.00 ± 11.55	c
	**4**	ND	c	70.00 ± 5.77	ab	100.00 ± 5.77	b	ND	d	73.33 ± 3.33	de	ND	c	193.33 ± 8.82	b
	**5**	ND	c	76.67 ± 3.33	a	136.67 ± 8.82	a	ND	d	43.33 ± 3.33	f	ND	c	385.00 ± 15.00	a
**AO**	**1**	ND	c	ND	f	ND	f	ND	d	ND	g	ND	c	ND	f
	**2**	ND	c	ND	f	ND	f	ND	d	ND	g	ND	c	ND	f
	**3**	86.67 ± 3.33	b	ND	f	ND	f	ND	d	ND	g	ND	c	ND	f
	**4**	110.00 ± 10.00	b	ND	f	ND	f	ND	d	ND	g	ND	c	ND	f
	**5**	2240.00 ± 40.00	a	ND	f	ND	f	ND	d	ND	g	ND	c	ND	f
		**Bioactive Compound Concentration (mg/g DW) ^1,2,3^**													
**Treatment**	**Fermentation Time (d)**	**N-L-γ-Glutamyl-S-p-Coumaryl-L-Cysteine**		**L-Tryptophan**		**Gallic Acid Glucoside**		**S-Coniferyl-L-Cysteine**		**N.I.**		**S-Sinapyl-L-Cysteine**		***p*** **-Coumaroyl-Feruloyl Glycerol**	
**Control**	**0**	78.00 ± 3.33	d	21.00 ± 1.45	c	ND	e	ND	g	ND	e	98.00 ± 4.55	c	27.05 ± 2.41	e
**LR**	**1**	ND	e	ND	d	80.00 ± 0.00	d	30.00 ± 0.00	f	ND	e	160.00 ± 5.77	ab	ND	f
	**2**	ND	e	ND	d	130.00 ± 11.55	c	190.00 ± 4.32	e	ND	e	170.00 ± 5.77	ab	ND	f
	**3**	ND	e	25.00 ± 10.61	c	130.00 ± 11.55	c	220.00 ± 15.28	e	ND	e	183.33 ± 13.33	a	ND	f
	**4**	ND	e	33.33 ± 8.82	bc	180.00 ± 5.77	b	242.67 ± 12.02	e	ND	e	150.00 ± 10.00	ab	ND	f
	**5**	ND	e	50.00 ± 0.00	ab	260.00 ± 5.77	a	325.00 ± 14.50	de	ND	e	165.00 ± 15.00	ab	ND	f
**LP**	**1**	206.67 ± 13.33	a	ND	d	ND	e	366.67 ± 17.64	d	ND	e	180.00 ± 5.77	a	280.00 ± 5.77	a
	**2**	146.67 ± 8.82	b	ND	d	ND	e	680.00 ± 25.17	c	ND	e	180.00 ± 15.28	a	170.00 ± 11.55	b
	**3**	106.67 ± 3.33	c	ND	d	ND	e	777.00 ± 3.00	c	ND	e	173.33 ± 12.02	ab	140.00 ± 10.00	c
	**4**	106.67 ± 8.82	c	50.00 ± 5.77	ab	ND	e	1353.00 ± 63.86	b	ND	e	163.33 ± 8.82	ab	90.00 ± 10.00	d
	**5**	115.00 ± 5.00	c	63.33 ± 3.33	ab	ND	e	1566.67 ± 83.73	a	ND	e	140.00 ± 20.00	ab	43.33 ± 8.82	e
**AO**	**1**	ND	e	ND	d	ND	e	ND	g	300.30 ± 10.00	d	ND	d	ND	f
	**2**	ND	e	ND	d	ND	e	ND	g	303.33 ± 8.82	d	ND	d	ND	f
	**3**	ND	e	ND	d	ND	e	ND	g	486.67 ± 8.82	b	ND	d	ND	f
	**4**	ND	e	ND	d	ND	e	ND	g	425.00 ± 5.00	c	ND	d	ND	f
	**5**	ND	e	ND	d	ND	e	ND	g	585.00 ± 5.00	a	ND	d	ND	f
		**Bioactive Compound Concentration (mg/g DW) ^1,2,3^**													
**Treatment**	**Fermentation Time (d)**	**Coumaric Acid Derivative**		**Ferulic Acid**		**Sinapic Acid**		**(di-E,E)-N,N´-Di Feruloyl Spermidine**		**N.I.**		**Feruloyl Hexoside**			
**Control**	**0**	ND	e	ND	h	ND	g	10.00 ± 0.02	c	ND	c	130.00 ± 8.16	d		
**LR**	**1**	ND	e	126.67 ± 3.33	f	ND	g	ND	d	ND	c	203.30 ± 6.67	c		
	**2**	ND	e	313.33 ± 3.33	e	ND	g	ND	d	ND	c	260.00 ± 15.28	b		
	**3**	ND	e	450.00 ± 11.55	c	ND	g	ND	d	ND	c	273.33 ± 6.67	b		
	**4**	ND	e	740.00 ± 30.00	b	ND	g	15.00 ± 0.50	c	ND	c	306.67 ± 23.33	b		
	**5**	ND	e	1415.00 ± 85.00	a	ND	g	40.00 ± 0.01	b	ND	c	356.67 ± 23.33	a		
**LP**	**1**	333.33 ± 18.56	d	20.00 ± 0.30	g	233.33 ± 8.82	e	30.00 ± 0.02	bc	193.33 ± 8.82	b	ND	e		
	**2**	496.67 ± 21.86	c	23.33 ± 3.33	g	326.67 ± 21.86	d	26.67 ± 3.33	bc	226.67 ± 12.02	b	ND	e		
	**3**	596.67 ± 35.28	b	26.67 ± 3.33	g	535.00 ± 15.00	c	26.67 ± 6.67	bc	240.00 ± 15.28	b	ND	e		
	**4**	785.00 ± 74.25	a	24.00 ± 2.10	g	756.67 ± 37.56	b	46.67 ± 8.82	ab	295.00 ± 5.00	a	ND	e		
	**5**	790.00 ± 70.00	a	26.67 ± 3.33	g	1075.00 ± 85.00	a	56.67 ± 12.02	ab	345.00 ± 28.50	a	ND	e		
**AO**	**1**	228.18 ± 10.00	d	395.00 ± 5.00	d	176.67 ± 6.67	ef	ND	d	ND	c	ND	e		
	**2**	323.33 ± 3.33	d	280.00 ± 10.00	e	143.33 ± 3.33	f	ND	d	ND	c	ND	e		
	**3**	373.33 ± 3.30	d	323.33 ± 3.30	e	156.67 ± 3.33	f	ND	d	ND	c	ND	e		
	**4**	295.00 ± 15.00	d	305.10 ± 5.00	e	146.67 ± 3.33	f	ND	d	ND	c	ND	e		
	**5**	335.00 ± 5.00	d	480.00 ± 10.00	c	243.33 ± 12.02	e	ND	d	ND	c	ND	e		

^1^ Concentrations are reported for each individual standard. Compounds were quantified at 280, 320, and 360 nm. Concentrations are expressed as equivalents of gallic acid, *p*-coumaric acid, ferulic acid, and quercetin. ^2^ Values represent the mean of three replicates ± standard error of the mean. ^3^ Different letters in the same column indicate statistical differences in the concentration of each compound between treatments using the least significant difference (LSD) test (*p* < 0.05). Abbreviations: LR: *L. rhamnosus*; LP: *L. plantarum*; AO: *A. oryzae*; N.I.: No identified; ND: Not detectable.

## Data Availability

The data used to support the findings of this study can be made available by the corresponding author upon request.

## Supplementary Materials

The following supporting information can be downloaded at: https://www.mdpi.com/article/10.3390/foods12224162/s1, Table S1: Chemical composition of pineapple peel; Figure S1: pH and Aw changes during solid-state fermentation of pineapple peels with *L. rhamnosus*, *L. plantarum*, and *A. oryzae*.
