# Supplementary material for: Solid-State Fermented Pineapple Peel: A Novel Food Ingredient with Antioxidant and Anti-Inflammatory Properties
Source: Foods. 2023 Nov 17;12(22):4162. doi: 10.3390/foods12224162 (PMC10670571; doi:10.3390/foods12224162)
Supplement: Supplementary file 1 [file foods-12-04162-s001.zip › foods-2707998-supplementary.pdf]

Solid-state fermented pineapple peel: a novel food ingredient with antioxidant and anti-inflammatory properties

Erika Ortega-Hernández [1,2,†], Lucio Martinez-Alvarado [1,†], Beatriz A. Acosta-Estrada [1,*] and Marilena Antunes-Ricardo [2,*]

[1] Tecnologico de Monterrey, Escuela de Ingeniería y Ciencias, Centro de Biotecnología-FEMSA, Ave. Eugenio Garza Sada 2501 Sur, Monterrey CP 64849, Mexico
[2] Tecnologico de Monterrey, Institute for Obesity Research, Ave. Eugenio Garza Sada 2501 Sur, Monterrey CP 64849, Mexico
* Correspondence: baae@tec.mx (B.A.A.-E.); marilena.antunes@tec.mx (M.A.-R.)
† These authors contributed equally to this work.

Supplementary Information

Table S1. Chemical composition of pineapple peel

Measured Parameter	Quantity	Units
Total reducing sugars	38.38 ± 0.59	%
Calories	208.85 ±	Kcal/100 g
Available carbohydrates	42.29 ±	%
Ashes	4.60 ± 0.13	%
Lipids	1.77 ± 0.04	%
Dietary fiber	30.20 ± 6.65	%
Moisture	15.2 ± 0.02	%
Protein	5.94 ± 0.03	%N x 6.25

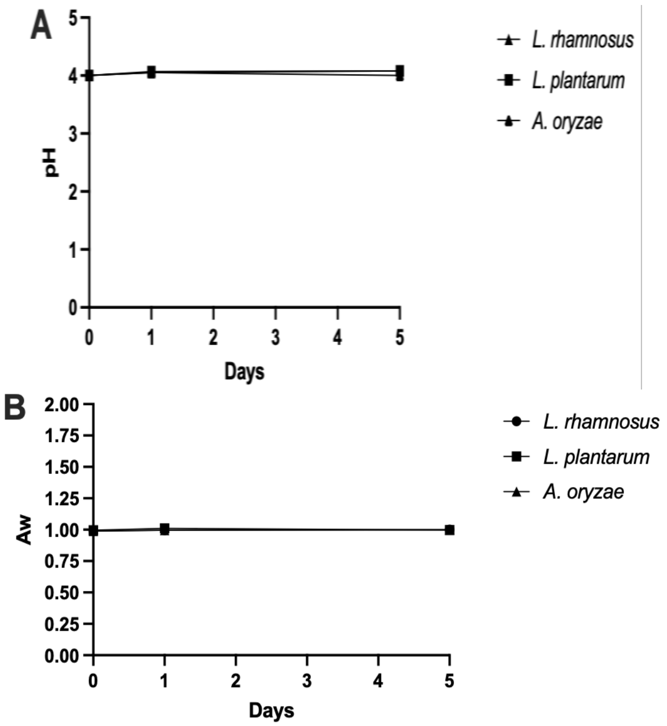

Figure S1. pH and Aw changes during solid-state fermentation of pineapple peels with *L. rhamnosus*, *L. plantarum* and *A. oryzae*. **A.** pH changes during solid-state fermentation of pineapple peels. **B.** Aw changes during solid-state fermentation of pineapple peels. Values represent the mean of 3 replicates with their standard error bars.
